# Production of polyunsaturated fatty acids in pork backfat fermented by *Mucor circinelloides*

**DOI:** 10.1007/s00253-024-13018-4

**Published:** 2024-02-20

**Authors:** Haisu Shi, Yingtong Zhang, Hao Lin, Yiran Yan, Ruhong Wang, Rina Wu, Junrui Wu

**Affiliations:** 1 College of Food Science, Shenyang Agricultural University, Shenyang, 110866 People’s Republic of China; 2 Liaoning Engineering Research Center of Food Fermentation Technology, Shenyang Agricultural University, Shenyang, 110866 People’s Republic of China; 3 Shenyang Key Laboratory of Microbial Fermentation Technology Innovation, Shenyang Agricultural University, Shenyang, 110866 People’s Republic of China; 4 Institute of Agricultural Facilities and Equipment, Jiangsu Academy of Agricultural Sciences, Nanjing, 210014 Jiangsu People’s Republic of China

**Keywords:** *Mucor circinelloides*, Ultrasound, Papain, Polyunsaturated fatty acids, Fermented pork backfat

## Abstract

**Abstract:**

Pork backfat (PB) contains excessive saturated fatty acids (SFAs), but lacks polyunsaturated fatty acids (PUFAs). Excessive SFAs can be used as a substrate for the growth of certain microorganisms that convert them into PUFAs and monounsaturated fatty acids (MUFAs), and the added value of PB can be enhanced. In this study, *Mucor circinelloides* CBS 277.49 and *Lactiplantacillus plantarum* CGMCC 24189 were co-cultured for conversion of PB into fermented pork backfat (FPB) with high level of PUFAs. Our results showed that the content of γ-linolenic acid (GLA) and linoleic acid (LA) in the surface of FPB reached 9.04 ± 0.14 mg/g and 107.31 ± 5.16 mg/g for 7-day fermentation, respectively. To convert the internal SFAs of PB, ultrasound combined with papain was used to promote the penetrative growth of *M. circinelloides* into the internal PB, and the GLA level in the third layer of fat reached 2.58 ± 0.31 mg/g FPB. The internal growth of *M. circinelloides* in PB was promoted by adjusting the oxygen rate and ventilation rate through the wind velocity sensor. When the oxygen rate is 2 m/s and the ventilation rate is 18 m^3^/h, the GLA level in the third layer of fat reached 4.13 ± 1.01 mg/g FPB. To further improve the level of PUFAs in PB, FPB was produced by *M. circinelloides* at 18 °C. The GLA content on the surface of FPB reached 15.73 ± 1.13 mg/g FPB, and the GLA yield in the second and third layers of fat reached 8.68 ± 1.77 mg/g FPB and 6.13 ± 1.28 mg/g FPB, the LA yield in the second and third layers of fat reached 105.45 ± 5.01 mg/g FPB and 98.46 ± 4.14 mg/g FPB, respectively. These results suggested that excessive SFAs in PB can be converted into PUFAs and provided a new technique for improving PUFAs in FPB.

**Key points:**

• *This article achieved the conversion of PUFAs in pork backfat by Mucor circinelloides CBS 277.49 and Lactiplantacillus plantarum CGMCC 24189.*

• *This article solved the internal growth of M. circinelloides CBS277.49 in pork backfat by ultrasound combined with papain.*

• *This article proposed an innovative of promoting the internal growth of M. circinelloides and increasing the PUFAs production by oxygen ventilation in pork backfat.*

**Supplementary Information:**

The online version contains supplementary material available at 10.1007/s00253-024-13018-4.

## Introduction

Pork backfat (PB) contains 90% lipids, among which saturated fatty acids (SFAs, mainly involving stearic acids and palmitic acids), monounsaturated fatty acids (MUFAs, mainly involving oleic acids), and polyunsaturated fatty acids (PUFAs, mainly involving linoleic acid (LA)) account for 40%, 46%, and 10% of total lipids, respectively. Lard can be refined from PB, and it can be used to produce shortening and margarine. However, excessive intake of SFAs is harmful for human health and may cause a series of chronic diseases such as obesity (Pinheiro-Castro et al. 2019), cardiovascular diseases (Spence et al. 2021), and metabolic diseases (German 2004; Nakamizo et al. 2018). Microbial fermentation of PB may solve this problem by improving the composition of fatty acids in meat products. Saturated fatty acids can be converted into PUFAs by certain microorganisms (such as *Mucor circinelloides* (Gaykawad et al. 2021), *Mortierella alpina* (Slany et al. 2020), *Mortierella isabelline* (Slany et al. 2021), *Geotrichum candidum* (Luo et al. 2019), *Yarrowia lipolytica* (Cotarlet et al. 2020), and *Lactiplantacillus plantarum* (Ozer and Kilic 2021)), thereby increasing the PUFA level of meat products.

*M. circinelloides* is a type of oleaginous fungus, which has high catalytic activity of fatty acid desaturases (FADS, including *FADS12*, *FADS6-1*, and *FADS6-2*) and accumulates γ-linolenic acid (GLA, C18:3, n-6). The activity of FADS and lipid accumulation in *M. circinelloides* can be affected by various cultural conditions, involving carbon source (Sun et al. 2021; Tzimorotas et al. 2018), nitrogen source (Dzurendova et al. 2020a), metal ions (Dzurendova et al. 2020b), temperature, and pH (Morin-Sardin et al. 2016). Hussain et al. (2019) found that it was important for the regulation of fatty acid synthesis in *M. circinelloides* using glucose combined with coconut oil as carbon source, and yeast extract as nitrogen source and at appropriate C/N ratio, which provides corresponding fatty acid precursors for FADS. In addition, different metal ions had shown strain-specific influence on fatty acid transformation in *M. circinelloides*. High levels of Zn^2+^/Mg^2+^ and low levels of Fe^3+^/Mn^2+^ can promote carbon metabolism flux of the pentose phosphate pathway and the glyoxylic acid cycle, which enabled the carbon source to be transformed directionally. Furthermore, fatty acid production of *M. circinelloides* was promoted at relatively low temperatures and weak acidity. The transcriptional level of the *FADS6* gene from *M. circinelloides* at 15 °C was rapidly upregulated and highly expressed, resulting in a 1.5-fold higher GLA yield than that at 28 °C (Michinaka et al. 2003).

Renewable sources of carbon and nitrogen sources of agricultural raw materials (Klempova et al. 2020; Zhang and Song 2021) or by-products of food industry (Braz et al. 2020; Tzimorotas et al. 2018) can be utilized by *M. circinelloides* to produce dietetic PUFAs. Mitra et al. (2012) reported that the PUFA level in *M. circinelloides* CBS 277.49 had been increased to 52% of total lipids by addition of thin stillage. In addition, *M. circinelloides* was utilized for GLA (Chan et al. 2018) production using whey as culture medium, and the GLA yield reached 464 mg/L (Chan et al. 2020). *M. circinelloides* was also used to convert vegetable oils (such as palm oil and soybean oil (Sun et al. 2021)) and emulsion hydrophobic substrates (prepared by animal fat (Gaykawad et al. 2021) and Tween containing specific fatty acid precursors) into PUFAs. So, it can be used for PUFA production in fermented foods with preferable quality, unique flavor, and texture properties. The milk clotting enzymes produced by *M. circinelloides* with addition of dhal husk can be used for the production of Cheddar cheese with a higher level of proteolysis and unique textures and tastes (Sathya et al. 2010). *Lactiplantacillus plantarum*, as a commonly used meat fermentation starter, can promote water loss and inhibit fat oxidation by acidifying and degrading carbohydrates in pork (Wang et al. 2019). The co-fermentation of *L. plantarum* and *M. circinelloides* enabled the starter penetrate into the interior more easily, thereby increasing the conversion rate of SFAs (Ge et al. 2019).

In this study, *M. circinelloides* CBS 277.49 and *L. plantarum* CGMCC 24189 were co-cultured for conversion of SFAs into PUFAs in PB, and the fermentation process was optimized to improve the LA and GLA level in FPB (fermented pork backfat). To convert the internal SFAs, PB samples were treated by ultrasound-assisted papain to promote the penetrative growth of *M. circinelloides* into the internal PB. By adjusting the oxygen rate and ventilation rate during fermentation, SFAs in the internal layers of PB were converted into PUFAs. To avoid excessive proteolysis by papain, PB samples were fermented under low temperature and the level of PUFAs was further improved.

## Materials and methods

### Preparation of spore suspension

*M. circinelloides* CBS 277.49 and *L. plantarum* CGMCC 24189 used in this study were obtained from our lab. The spore suspensions for inoculation were prepared from a 14-day-old culture grown on potato dextrose agar (PDA) medium. Spores have been washed using an aqueous solution of 0.9% saline, and the suspension was diluted to a final concentration of 1.0 × 10^7^ spores/mL. The *L. plantarum* culture for inoculation was prepared from a 14-day-old culture grown on DeMan-Rogosa-Sharpe (MRS) medium. After incubated at 37 °C for 1 day, the concentration was adjusted to 1.0 × 10^7^ cells/mL.

### Conditions of PB fermentation

The surface of meat batter and meat chunk containing 30 g of subcutaneous PB in a sterile Petri dish was inoculated with 2 mL of spore suspensions (the ratio of *M. circinelloides* CBS 277.49 to *L. planturum* CGMCC 24189 was 3:1) on the surface of meat at 30 °C for 7 days. To investigate the culture conditions on the level of PUFAs in meat fermentation, the cultures were incubated at different temperatures (15 °C, 18 °C, 21 °C, 24 °C, 27 °C), different culture times (3, 5, 7, 9, 11 days), and different inoculation (1%, 1.5%, 2.0%, 2.5%, 3.0%, *v/v*). All experiments were performed in three independent biological replicates.

### Analysis of fatty acid profiles in FPB

The total lipids was extracted from 30 g of the fermented meat, according to the procedure by Folch et al. (1957), and 50 mg of fat was used to determine the fatty acid profiles. Fatty acids from PB fermentation matrixes were converted into their fatty acid methyl esters (FAMEs) by a modified method of Teixeira et al. (2020); 4 mL of hexane was added and vortexed every 2 min for 5 min at room temperature; then, 4 mL 0.4 mol/L KOH/methanol was vortexed briefly, and samples were incubated at 50 °C for 1 h before adding 2 mL of distilled water. The organic phase (with the methyl esters of fatty acids) was extracted with 2.5 mL of hexane. FAMEs were subsequently analyzed by gas chromatography according to the method described by Čertík et al. (2013).

### Ultrasonic and papain pretreatment of PB

Each meat sample was cut into pieces 3 × 3 × 3 cm in dimension, and sealed separately, labeled, and kept frozen at − 18 °C. The samples were divided in two groups: one group was treated with ultrasound (100 W, 320 W) for 20 min and another group of papain (0.1, 0.2, 0.3, 0.4, 0.5 g/100 mL) for 25 min and ultrasound (100 W, 320 W) for 20-min treatment. All the treatments were carried out as described in Barekat and Soltanizadeh (2019). A wind velocity sensor was placed during the middle and late stages of fermentation (3–7 days) to adjust the oxygen rate (1, 1.5, 2, 2.5, 3 m/s) and ventilation rate (ventilation pipe radius of 2, 2.5, 3, 3.5, 4 cm). Meat samples were divided into three pieces with a thickness of 0.5 cm in each piece (designated first layer, second layer, and third layer from the depth of 0–0.5 cm, 0.5–1 cm, and 1–1.5 cm of the sample surface, respectively), and the fatty acids, texture characteristics, and shear force were determined.

### Texture analysis

The texture was analyzed using a texture analyzer; the Warner-Bratzler shear force (WBSF) measurement was performed on meat samples according to Saengsuk et al. (2021).

For texture profile analysis (TPA), PB samples treated with ultrasonic and papain were analyzed according to Barekat and Soltanizadeh (2019). For all the analysis, the samples were compressed perpendicular to the direction of muscle fibers using a cylindrical probe with 13-mm diameter (Barekat and Soltanizadeh 2017b).

### Inverted fluorescence microscopy analysis

The appropriate thin meat slices were cut longitudinally from the fermented meat block with a blade and placed on a glass slide, placed in an oven at 60 °C until the meat slices were taken out in a transparent state, and placed in a 10% phosphate buffer (pH 6.9–7.1). The surface lipid was removed using chloroform solution by 2–3 repeats. The dried contact piece was fixed with methanol for 1 min, and the lactic acid carbolic acid cotton blue dyeing solution staining solution was placed for 3–5 min. The snapshots of fermentation products were obtained by inverted fluorescence microscope TS100-F (Shenyang Hejun Biotechnology Co., Ltd., China).

### The culture of M. circinelloides at low temperature

*M. circinelloides* CBS277.49 cells were cultured in a 250-mL flask with PDA medium by inoculation of 1 × 10^7^ cell mL^−1^ at 15 °C, 18 °C, 21 °C, 24 °C, and 27 °C, respectively. There were 3 replicates at each sample. After 24 h of culture at different temperatures, 100 mL cultures were centrifuged at 6000 r/min for 5 min, and the wet cells obtained by washing and centrifugation with distilled water were repeated twice. The dried weighing bottle and wet cells were placed in a 50 °C oven to dry to constant weight, and the biomass was calculated. To investigate the effect of temperature on lipid contents and the transcriptional level of desaturase genes, 200 mL cultures were taken from each sample for real-time quantitative PCR analysis and 100 mL aliquots for fatty acid analysis.

### RNA isolation and RT-qPCR analysis

Approximately 100 mg (dry weight) of *M. circinelloides* CBS277.49 tissue was ground to a fine powder with a precooled mortar and pestle using liquid nitrogen. Total RNA was isolated using the BIOG RNA Fungi & Bacteria Kit (Changzhou Baidai Biotechnology Co., Ltd., China), and reverse-transcribed with NovoScript® (Suzhou Inshore Protein Technology Co., Ltd., China) Plus All-in-One First-Strand cDNA Synthesis Supersix as described previously (Shi et al. 2016). Three pairs of specific primers for RT-PCR were designed according to our previous work (Shi et al. 2020) (Table 1) and sequence information from the NCBI database. RT-PCR was performed according to the procedure by Caramalho et al. (2019). Relative expression of desaturase gene products was normalized to the housekeeping gene 18S rRNA and calculated using the 2^−ΔΔCT^ method (Damgaard and Treebak 2022).

**Table 1:** Sequences of primers used in this study

Primer names	Nucleotide sequences (5′-3′)
FADS12 q-F	GCCCACATCAAGAAGGCTCT
FADS12q-R	CTTCGACAAAGCGACAGCTC
FADS6-1 q-F	GGCTAATTGCTACGTTGGCG
FADS6-1 q-R	TACACACAAGCAGGGTGGTC
FADS6-2 q-R	TTGACCAAAGGCACGCATTG
FADS6-2q-F	ATGTGGATTGCCCAGAGTGG
18S r RNA-qF	ACTTCACCGTGCTGGGGATA
18S r RNA-qR	CAAAGGGCAGGGACGTAATC

*FADS12* Δ12-fatty acid desaturase, *FADS6-1 and FADS6-2* Δ6-fatty acid desaturase isozymes

### Fatty acid extraction and analysis

Determination of lipid content and fatty acid composition of fungal biomass was performed by direct transesterification method according to Tan et al. (2017).

### Statistical analysis

All experiments were conducted in triplicate unless specified. Statistical analysis was evaluated by ANOVA calculated in SPSS 26.0 (IBM, Armonk, NY, USA). Origin 2022 (OriginLab, Northampton, MA, USA) was used to plot the data and analysis. Differences were regarded as significant at *P* value and expressed as the mean ± standard deviation (SD). The significance at *P* < 0.05 was evaluated by Tukey’s test.

## Results

### Conversion of PUFAs in PB by mixed fermentation

**Fig. 1:**
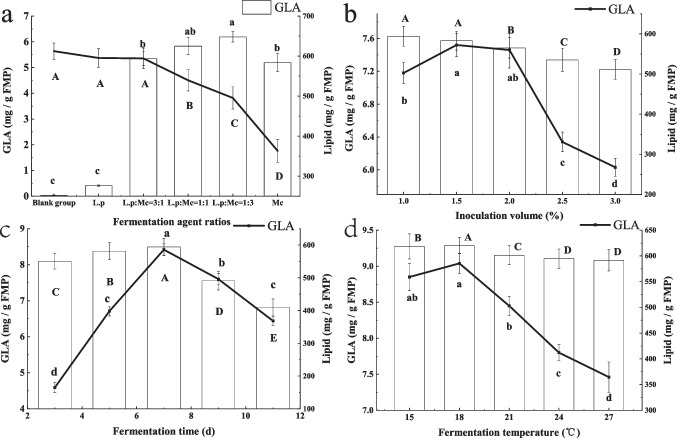
Effect of fermentation conditions on GLA and lipid level in FPB. **A** Starter ratios. **B** Inoculation volume (*v/v*). **C** Fermentation time. **D** Fermentation temperature. Dissimilar letters indicate significant difference (*P* < 0.05)

To convert SFAs into PUFAs in PB, *M. circinelloides* CBS 277.49 and *L. plantarum* CGMCC 24189 were used in pork fermentation. Our result showed that the GLA level in PB reached 6.19 ± 0.12 mg/g FPB by a 3:1 inoculation proportion of *M. circinelloides* CBS 277.49/*L. plantarum* CGMCC 24189, resulting in a 1.19-fold increase compared to the control (0.03 ± 0.02 mg/g FPB) (Fig. 1a). The yield of GLA increased to 7.51 ± 0.14 mg/g FPB at 1.5% inoculation of *M. circinelloides* CBS 277.49 and *L. plantarum* CGMCC 24189 (Fig. 1b). The highest GLA and LA level reached 9.04 ± 0.14 mg/g FPB and 107.31 ± 5.16 mg/g FPB for 7 days at 18 °C, respectively, and the yield of GLA was 1.62-fold higher than that in the control (Fig. 1c, d).

### Conversion of PUFAs in the internal layers of PB by ultrasound

**Fig. 2:**
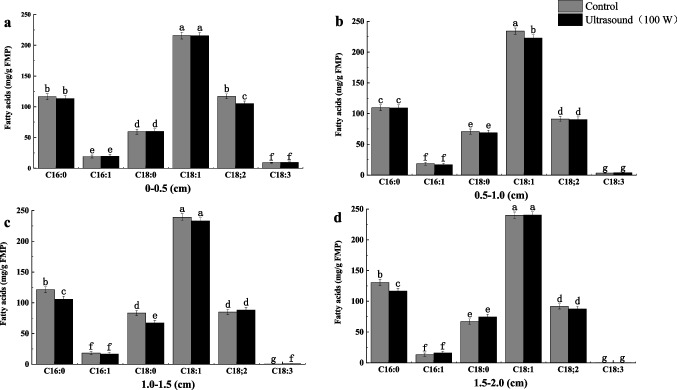
Effect of ultrasound on fatty acid composition in FPB. Error bars represent the average ± SD of three separate experiments. 0–0.5 cm represents the first layer; 0.5–1.0 cm represents the second layer; 1.0–1.5 cm represents the third layer; 1.5–2.0 cm represents the fourth layer. Dissimilar letters indicate significant difference (*P* < 0.05)

*M. circinelloides* generally grows on the surface of meat, and external lipids can be converted, but the internal layers of PB are difficult to be utilized. To solve this bottleneck, ultrasound treatment on meat cells was performed to promote the penetration of microbes, which is conducive to conversion of the internal fatty acids of PB. The results showed that ultrasound-induced cavitation caused physical damage to meat. The hardness and shear force in the third layer of PB decreased by 14.12% and 20.73% at the 100 W of ultrasonic power for 20 min (Table 2), respectively. It facilitated *M. circinelloides* to grow in the deeper layer of PB and convert the internal layers of PUFAs. The yield of GLA and LA in the third layer reached 1.05 ± 0.18 mg/g FPB and 88.28 ± 4.36 mg/g FPB, respectively, whereas the GLA yield sharply decreased to just 0.13 ± 0.02 mg/g FPB in the fourth layer (1.5–2.0 cm) of FPB (Fig. 2).

**Table 2:** Effects of ultrasound-assisted papain on texture characteristics, and shear force in each layer of PB

	The layer of PB (cm)	Hardness (N)	Elasticity (nm)	Shear force (N)
Control group	0–0.5	20.01 ± 0.12 a	0.53 ± 0.13 a	17.42 ± 0.11 a
	0.5–1.0	16.22 ± 0.13 b	0.53 ± 0.11 a	14.04 ± 0.13 b
	1.0–1.5	14.87 ± 0.21 c	0.52 ± 0.18 a	12.54 ± 0.16 c
Ultrasound (100 W)	0–0.5	17.47 ± 0.14 b	0.45 ± 0.12 a	15.41 ± 0.11 b
	0.5–1.0	15.67 ± 0.22 bc	0.42 ± 0.17 a	12.87 ± 0.13 bc
	1.0–1.5	11.22 ± 0.16 d	0.42 ± 0.14 a	10.94 ± 0.16 cd
Ultrasonic (100 W)-assisted papain (0.3%)	0–0.5	7.60 ± 0.13 e	0.42 ± 0.11 a	6.39 ± 0.14 d
	0.5–1.0	5.09 ± 0.11 f	0.36 ± 0.14 ab	4.14 ± 0.09 de
	1.0–1.5	4.32 ± 0.13 f	0.32 ± 0.12 b	3.83 ± 0.14 e

Error bars represent the average ± SD of three separate experiments. Different letters in each column indicates significant difference between treatments (*P* < 0.05)

### Conversion of PUFAs in the internal layers of PB by papain

**Fig. 3:**
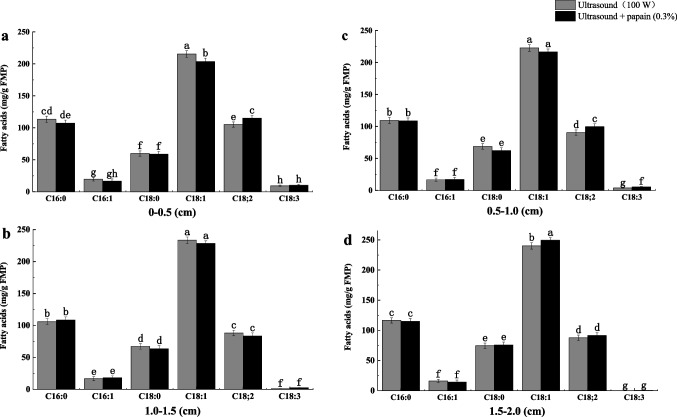
Effect of ultrasound and papain treatment on fatty acid composition of FPB. Dissimilar letters indicate significant difference (*P* < 0.05)

To further promote *M. circinelloides* CBS277.49 to penetrate in the internal growth of meat, papain was used to decompose myofibrillar protein and connective tissue protein in meat. With ultrasonic (100 W) assisted papain (0.3%) treatment, the hardness and shear force in the third layer were decreased by 61.49% and 69.45%, respectively, compared to the ultrasound group (Table 2). Our result found that the GLA content was significantly increased in the first, second, and third layers by 1.08-, 1.46-, and 2.46-fold, compared with the ultrasonic-treated group (Fig. 3). The LA yield in the third layer decreased from 91.59 ± 4.17 to 83.90 ± 4.19 mg/g FPB. In addition, the yield of GLA decreased to just 0.40 ± 0.11 mg/g FPB in the fourth layer (1.5–2.0 cm), owing to lack of oxygen content in the deeper layer of the meat. Thus, the optimal penetration depth of *M. circinelloides* CBS277.49 growth reached 1.0–1.5 cm of the meat.

The shape of myofibrillar protein and connective tissue was the same, the size was uniform, and the gap was narrow (Supplemental Fig. S1a). By papain (0.3 g/100 mL, 55 °C, 25 min) and ultrasonic (100 W, 20 min) treatment, the tissue was damaged and the gap became larger (Supplemental Fig. S1b). The penetration depth of *M. circinelloides* mycelium reached 1.22–1.64 cm of the meat (Supplemental Fig. S1c, d).

### Conversion of PUFAs in the internal layers of PB by ventilation

**Fig. 4:**
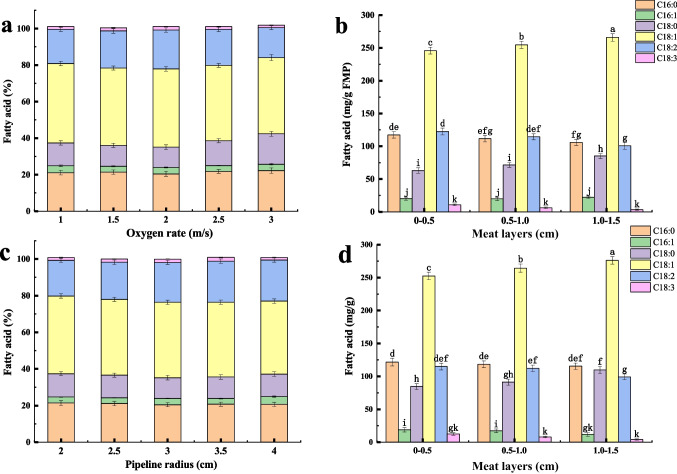
Effect of oxygen rate on fatty acid content in FPB. **A** Effect of different oxygen rates on fatty acids from the first layer of FPB. **B** Effect of oxygen rate (2 m/s) on fatty acid composition in different layers of FPB. **C** Effect of different pipeline radius on fatty acid composition in the first layer of FPB. **D** Effect of pipeline radius (3 cm) on fatty acid levels in each layer of FPB. Ventilation rate (m^3^/h) = wind speed (m/s) × pipeline cross-sectional area (m^2^) × 3600. Dissimilar letters indicate significant difference (*P* < 0.05)

To enhance the oxygen content in the deeper layer of the meat, the wind speed sensor system was used for supplying normal air, which penetrated *M. circinelloides* mycelia into the internal layers of the meat and promoted PUFA production in FPB. The results showed that the yield of PUFAs in the first layer of FPB increased from 17.77 to 23.21%. The highest ratio of PUFAs to SFAs reached 1.22:1.59 at 2 m/s oxygen rate (Fig. 4a), at which the yields of LA and GLA reached 101.83 ± 4.74 mg/g FPB and 3.36 ± 1.01 mg/g FPB in the third layer of FPB, respectively (Fig. 4b).

At the oxygen rate of 2 m/s, different radius of aeration pipe was used to increase the ventilation, which promoted the growth of *M. circinelloides* into the surface and the internal of PB. As shown in Fig. 4c, the ventilation rate reached 27.69 m^3^/h in a 3.5-cm-diameter pipe. The proportion of unsaturated fatty acids in the first layer of FPB reached 24.54%, and the yield of GLA reached 12.56 ± 2.11 mg/g FPB. LA and GLA yields reached 99.23 ± 4.36 mg/g FPB and 4.16± 1.03 mg/g FPB in the third layer of FPB, respectively (Fig. 4d).

### Effects of low temperature on PUFA production in FPB

**Fig. 5:**
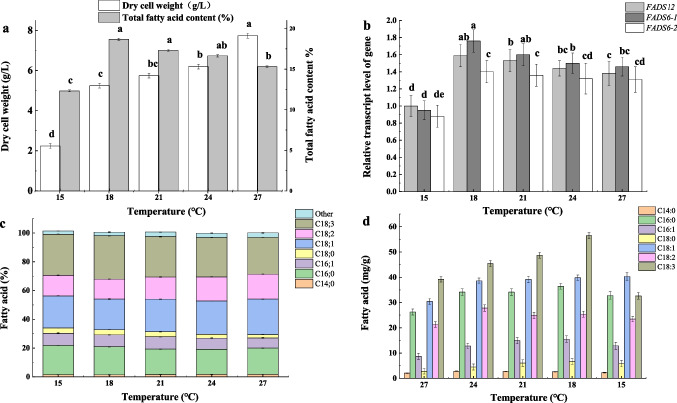
Effects of different temperatures on PUFA production in FPB. **A** Dry cell weight and total fatty acid content. **B** Relative transcript levels of *FADS12*, *FADS6-1*, and *FADS6-2* gene. The relative transcript levels were calculated using the 2^−ΔΔCt^ method. **C** Effect of temperature on fatty acid composition in the first layer of FPB by *M. circinelloides* CBS2777.49. **D** Effect of temperature on fatty acid levels in the first layer of FPB by *M. circinelloides* CBS2777.49. Different letters indicate significant difference (*P* < 0.05). Dissimilar letters indicate significant difference (*P* < 0.05)

To ensure high activity of fatty acid desaturase system for enhancing the PUFA production by *M. circinelloides* and to avoid excessive proteolysis by papain, PB was fermented at low temperature under the condition of ultrasound-assisted enzyme for 45 min (100 W + 0.3% papain), the oxygen rate (2 m/s), and ventilation rate (27.69 m^3^/h). As shown in Fig. 5a, the lipid yield of *M. circinelloides* CBS2777.49 cultured at 18 °C reached 18.68%. The expression level of the *FADS12*, *FADS6-1*, and *FADS6-2* genes in *M. circinelloides* CBS2777.49 was analyzed at different temperatures (Fig. 5b). The results showed that the expression level of *FADS6-1* at 18 °C was 1.21-fold higher than that at 27 °C, whereas the relative expression level of *FADS6-2* was just 0.95. High activities of FADS12 and FADS6 promoted the production of PUFAs, reaching 44.03% at 18 °C (Fig. 5c), and the GLA yield reached 56.41± 1.22 mg/g, 43.80% higher than that at 27 °C (Fig. 5d).

**Fig. 6:**
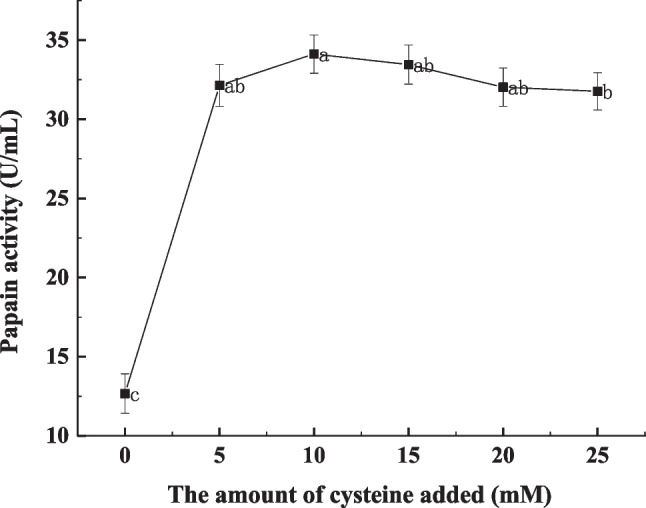
Effects of different levels of L-cysteine on papain activity during ultrasound (100 W, 18 °C). Dissimilar letters indicate significant difference (*P* < 0.05)

**Fig. 7:**
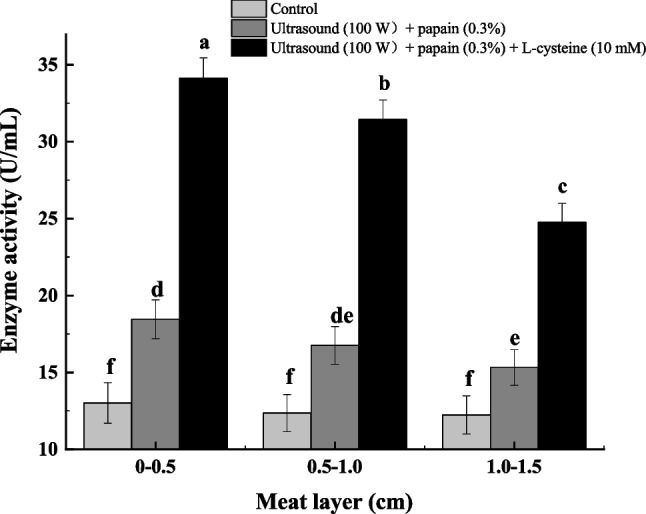
Protease activity in each layer of meat treated with L-cysteine papain solution combined with ultrasound (100 W, 18 °C). Dissimilar letters indicate significant difference (*P* < 0.05)

**Fig. 8:**
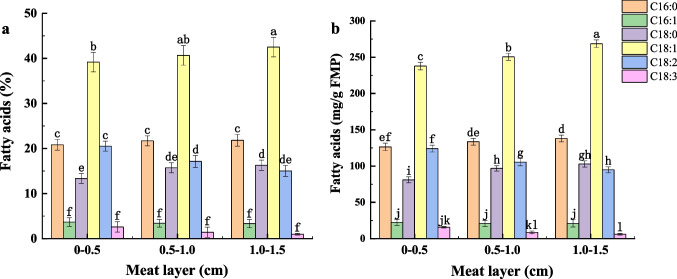
Fatty acid composition in each meat layers of FPB by inoculation of *M. circinelloides* in a L-cysteine papain solution treated with ultrasound (100 W, 18 °C). **A** Fatty acid composition. **B** Fatty acid level. Dissimilar letters indicate significant difference (*P* < 0.05)

Although low temperature reduced excessive myofibrillar proteolysis and increased the PUFA level in FPB, it influenced the depth of channel for *M. circinelloides* into the internal of the meat. L-Cysteine can be used with papain, which enabled to improve the activity and stability of the papain at low temperature (Sluyterman 1967). By addition of 10 mM L-cysteine combined with ultrasonic treatment (100 W, 18 °C) of PB, the papain activity reached 34.14 ± 1.19 U/mL, and the protease activity in the third layer of meat reached 25.08 ± 0.97 U/mL (Fig. 6). Compared with untreated and ultrasound-assisted papain treatment, the total protease activity in PB increased by 2.04-fold and 1.60-fold, respectively (Fig. 7). By the optimal treatment (ultrasound + L-cysteine + papain), *M. circinelloides* CBS277.49 was used to convert PUFAs in PB at 18 °C. The results showed that the composition of PUFAs in the first and third layers of FPB reached 23.45% and 16.01%, respectively (Fig. 8a). The content of GLA in the first layer of FPB reached 15.75 ± 1.11 mg/g FPB, which was 22.68% higher than that in the first layer of FPB treated by ultrasound-assisted papain. The content of GLA in the second and third layers of FPB reached 8.69 ± 1.75 mg/g FPB and 6.14 ± 1.26 mg/g FPB, respectively, whereas the content of LA in the second and third layers of FPB reached 105.45 ± 5.01 mg/g FPB and 94.90 ± 4.19 mg/g FPB, respectively (Fig. 8b).

## Discussion

Dietary intake of PB containing excessive saturated fat may cause cardiovascular disease (Zimmerman et al. 2021). Conversion of excessive saturated fats into PUFAs is an effective way to increase the added value of PB. Besides improving the flavor (Cai et al. 2020), quality (Copetti 2019), and shelf life (Ashaolu et al. 2021) in FPB, main starters and adjunct starters (Ashaolu et al. 2021; Lu et al. 2021) can be also used as conversion factories of PUFAs (Arief et al. 2016; Ashaolu et al. 2021; Čertík et al. 2013; Cotarlet et al. 2020). *M. circinelloides* CBS277.49 contains an efficient fatty acid desaturase system for PUFA production (Vasiliadou et al. 2018). However, the internal fatty acids in PB cannot be utilized by *M. circinelloides*, owing to the lack of oxygen among the internal meat cells for the growth of mycelium. By ultrasound-assisted papain, intercellular space among meat tissue had been expanded, and oxygen was supplied into the tissue gap for the growth of *M. circinelloides* and the conversion of PUFAs.

Ultrasound combined with papain can effectively expand the gap among the cells of meat. The cavitation enhances the ventilation permeability and increases the rate at which papain can penetrate the internal of the meat. Ultrasound promotes rapid papain to the “Z” line level of the fibrin heavy chain (Barekat and Soltanizadeh 2017a; Maqsood et al. 2018) while exploiting papain to have some targeted effects on myofibrillar protein and myosin heavy chain (Zhao et al. 2012). By adjusting the external environment to determine its enzymatic kinetic properties (Bekhit et al. 2014) and synergizing with endogenous proteases in meat to degrade myofibril tissue and lyse connective tissue, it can increase the index of myofibril fragmentation and decrease the particle size of myofibril protein (Supplemental Fig. S2). Maqsood et al. (2018) reported that papain had a significant effect on the breakdown of various proteins in camel meat, including myosin heavy chains, with a degradation rate of up to 22.4%. Combined with ultrasonic treatment of PB, the diffusion of papain in PB can be enhanced (Barekat and Soltanizadeh 2017a). Zhang et al. (2023) indicated the effects of lactic acid, ultrasound, and papain on beef, which increased the myofibril fragmentation index by 4.3 times. Thus, ultrasound-assisted papain can significantly break the integrity of PB, denature its myofibrillar proteins, and change the internal spatial structure of PB, in order to infiltrate the growth of *M. circinelloides* CBS277.49 in FPB and transform SFAs into PUFAs.

*M. circinelloides* CBS277.49 acts as an aerobic fungus, and oxygen delivery can effectively improve its biomass and lipid transformation. After the meat was treated by ultrasound-assisted papain, venting and fermentation reduced the water-holding capacity of the meat, providing a favorable environment for PUFA transformation in *M. circinelloides* CBS277.49. The increase of the oxygen rate is beneficial to the penetration of the mycelia along the myogenic gap, and the increase of the ventilation rate can increase the oxygen content per unit area of the FPB, which promoted the mycelia to further penetrate along the pores. When the oxygen rate and the ventilation rate reached 2.5 m/s and 36.17 m^3^/h, the surface mycelia are easy to be blown away, and the transformation of PUFAs in deeper layers can be inhibited.

Fermentation temperature is the key factor to influence the production of PUFAs in FPB. At higher fermentation temperature, the activity of papain can be reduced and even the formation of aggregates can be induced by prolonged fermentation (Li et al. 2021). Low temperature can effectively maintain the activity of proteases, degrade myofibril protein and collagen in connective tissue, and increase myofibril fragmentation index (Zhao et al. 2012; Zheng et al. 2022). However, oxidation of the sulfhydryl group in the active center of papain under prolonged low-temperature condition at 18 °C can be oxidated, and papain activity can be decreased (Grzonka et al. 2001), which inhibited the conversion of internal fat in PB by *M. circinelloides* CBS277.49 and affected the production of PUFAs. As an activator, L-cysteine played a key role in restoring papain activity by converting the sulfhydryl group from the oxidized (-S-S-) to the reduced (-SH-) state at low temperature (Sluyterman 1967; Storer and Menard 1994), thus promoting the release of the N-terminal fragment of papain and improving the affinity for the binding site of papain in the P2 position of the substrate (Bekhit et al. 2014). In addition, *L. plantarum* provided a slightly acidic environment for papain in meat, which remained the stability and activity of papain at low temperature, resulting in a loosely arranged structure in PB, with random folding of myofibrillar proteins leading to some unfolding and stretching, and the formation of larger gaps (Sluyterman 1967).

In this study, *M. circinelloides* and *L. plantarum* were co-cultured for conversion of PB into high value-added FPB with high level of PUFAs. To produce PUFAs in PB, the fermentation conditions were optimized, and ultrasonic-assisted papain was used to effectively promote the penetration of *M. circinelloides* mycelia into the intercellular space of PB for enhancing the conversion rate of PUFAs in FPB. PB was fermented by *M. circinelloides* at low temperature for further improving PUFAs level in FPB.

## Supplementary information

Supplementary file 1

## Data Availability

Data sharing is not applicable to this article as no datasets were generated or analyzed for this article.
