# Supplementary material for: Production of polyunsaturated fatty acids in pork backfat fermented by Mucor circinelloides
Source: Appl Microbiol Biotechnol. 2024 Feb 20;108:223. doi: 10.1007/s00253-024-13018-4 (PMC10879235; doi:10.1007/s00253-024-13018-4)
Supplement: Supplementary file 1 [file 253_2024_13018_MOESM1_ESM.pdf]

Supplementary Materials

Journal name:

Applied Microbiology and Biotechnology,

Manuscript Title:

Production of polyunsaturated fatty acids in pork backfat fermented by *Mucor circinelloides*

The names of the authors:

Haisu Shi, Yingtong Zhang, Hao Lin, Yiran Yan, Ruhong Wang, Rina Wu and Junrui Wu.

The affiliations and addresses of the authors:

Haisu Shi:

College of Food Science, Shenyang Agricultural University, Shenyang 110866, P.R. China

E-mail: shihaisu@syau.edu.cn

Yingtong Zhang:

Institute of Agricultural Facilities and Equipment, Jiangsu Academy of Agricultural Sciences, Nanjing 210014, Jiangsu, P.R. China

E-mail: ytzhang@jaas.ac.cn

Hao Lin:

College of Food Science, Shenyang Agricultural University, Shenyang 110866, P.R. China

E-mail: linhao212113@163.com

Yiran Yan:

College of Food Science, Shenyang Agricultural University, Shenyang 110866, P.R. China

E-mail: yanyiran0708@163.com

Ruhong Wang:

College of Food Science, Shenyang Agricultural University, Shenyang 110866, P.R. China

E-mail: wrh19980615@163.com

The e-mail address, telephone and fax numbers of the corresponding author

Rina Wu

College of Food Science, Shenyang Agricultural University, Shenyang 110866, P.R. China

Liaoning Engineering Research Center of Food Fermentation Technology, Shenyang Agricultural University, Shenyang 110866, P.R. China

Shenyang Key Laboratory of Microbial Fermentation Technology Innovation, Shenyang Agricultural University, Shenyang 110866, P.R. China

Telephone: (86)024-88487161

E-mail: wrn6956@163.com

Junrui Wu

College of Food Science, Shenyang Agricultural University, Shenyang 110866, P.R. China

Telephone: (86)024-88487161

E-mail: junruiwu@126.com

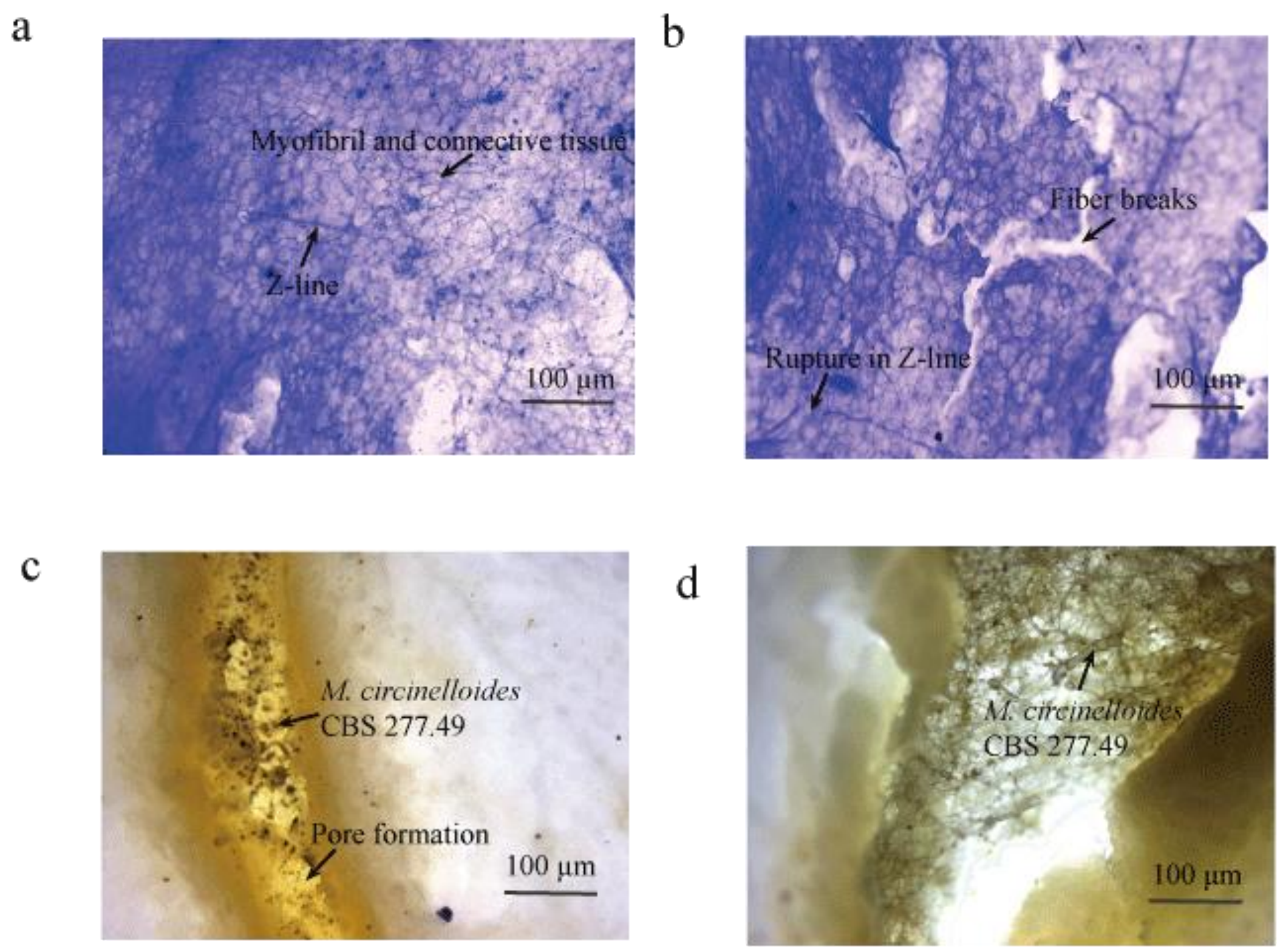

Figure S1 Inverted fluorescence microscopy analysis. A: complete myofibrillar tissue in untreated PB; B: the myofibrillar tissue of PB was treated by ultrasonic power of 100W, at 55°C, papain content of 0.3 % aqueous solution for 45 min; C: the myofibrillar tissue of the second layer of FPB (0.5-1.0 cm) under the inverted fluorescence microscope at 10 ×; D: the myofibrillar tissue of the third layer of FPB (1.0-1.5 cm) under the inverted fluorescence microscope at 10 ×.

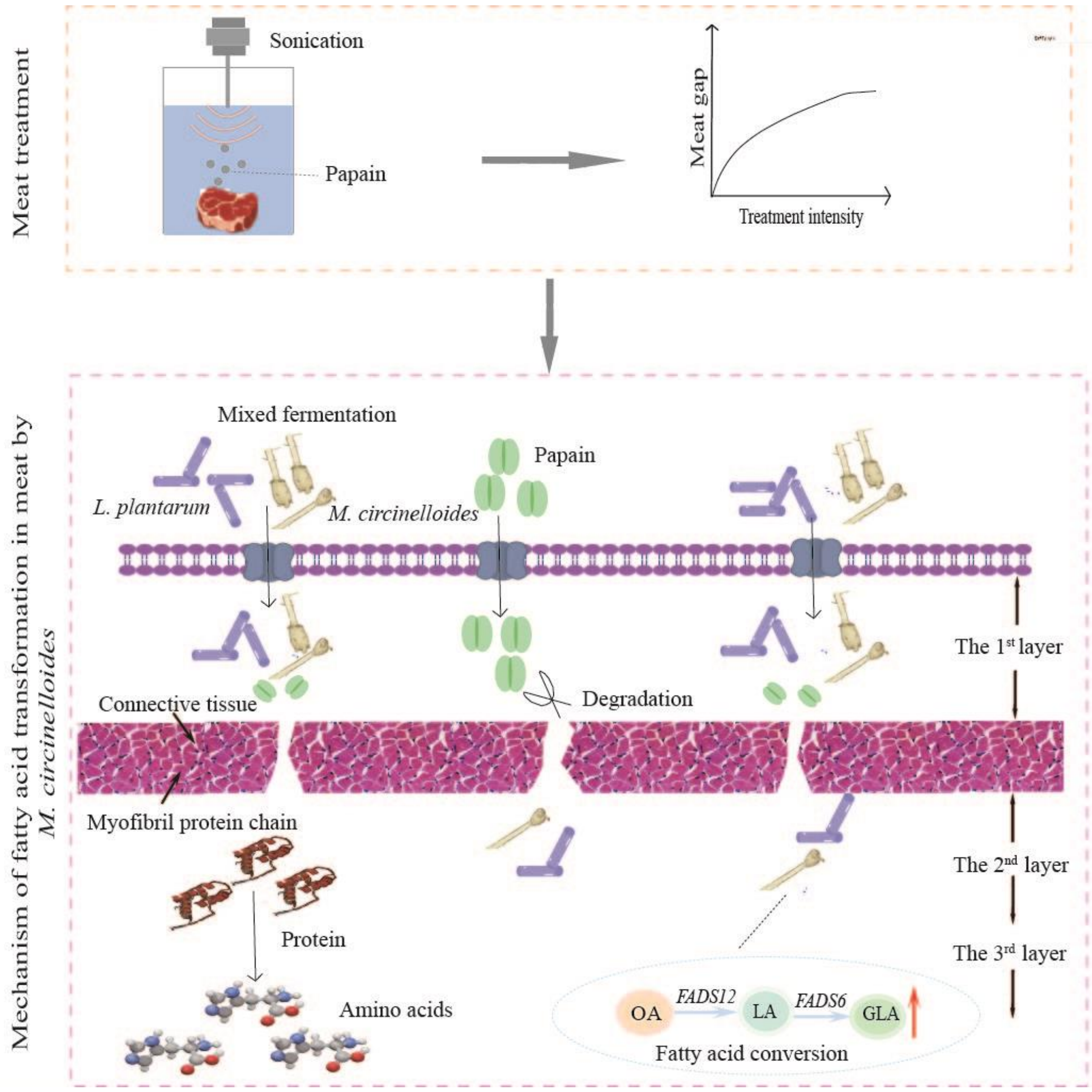

Figure S2 Schematic map of the effect of ultrasound-assisted papain on the internal meat tissue and the penetration process of *M. circinelloides* and *L. plantarum* into the internal meat tissue.
